# Autoimmunity as an Etiological Factor of Cancer: The Transformative Potential of Chronic Type 2 Inflammation

**DOI:** 10.3389/fcell.2021.664305

**Published:** 2021-06-21

**Authors:** Chris M. Li, Zhibin Chen

**Affiliations:** ^1^Department of Microbiology and Immunology, University of Miami Miller School of Medicine, Miami, FL, United States; ^2^Sylvester Comprehensive Cancer Center, University of Miami Miller School of Medicine, Miami, FL, United States

**Keywords:** autoimmunity, type 2 immunity, interleukin-4, interleukin-13, chronic inflammation, tumorigenesis, metaplasia, cancer

## Abstract

Recent epidemiological studies have found an alarming trend of increased cancer incidence in adults younger than 50 years of age and projected a substantial rise in cancer incidence over the next 10 years in this age group. This trend was exemplified in the incidence of non-cardia gastric cancer and its disproportionate impact on non-Hispanic white females under the age of 50. The trend is concurrent with the increasing incidence of autoimmune diseases in industrialized countries, suggesting a causal link between the two. While autoimmunity has been suspected to be a risk factor for some cancers, the exact mechanisms underlying the connection between autoimmunity and cancer remain unclear and are often controversial. The link has been attributed to several mediators such as immune suppression, infection, diet, environment, or, perhaps most plausibly, chronic inflammation because of its well-recognized role in tumorigenesis. In that regard, autoimmune conditions are common causes of chronic inflammation and may trigger repetitive cycles of antigen-specific cell damage, tissue regeneration, and wound healing. Illustrating the connection between autoimmune diseases and cancer are patients who have an increased risk of cancer development associated with genetically predisposed insufficiency of cytotoxic T lymphocyte-associated protein 4 (CTLA4), a prototypical immune checkpoint against autoimmunity and one of the main targets of cancer immune therapy. The tumorigenic process triggered by CTLA4 insufficiency has been shown in a mouse model to be dependent on the type 2 cytokines interleukin-4 (IL4) and interleukin-13 (IL13). In this type 2 inflammatory milieu, crosstalk with type 2 immune cells may initiate epigenetic reprogramming of epithelial cells, leading to a metaplastic differentiation and eventually malignant transformation even in the absence of classical oncogenic mutations. Those findings complement a large body of evidence for type 1, type 3, or other inflammatory mediators in inflammatory tumorigenesis. This review addresses the potential of autoimmunity as a causal factor for tumorigenesis, the underlying inflammatory mechanisms that may vary depending on host-environment variations, and implications to cancer prevention and immunotherapy.

## Introduction: Autoimmunity Is Emerging as a Risk Factor for Cancer Development in Young Adults

Gastric cancer is the third leading cause of cancer death globally, with a 5-year survival rate of 5.3% for patients with advanced diseases (Chandra et al., 2020). Typically, gastric cancer is 2.2 times more likely to be diagnosed in males in developed countries (Rawla and Barsouk, 2019) and is strongly associated with *Helicobacter pylori* infection (Correa, 2013). While the overall incidence of gastric cancer has been decreasing, which can likely be attributed to decreased incidence of *H. pylori* infection (Blaser and Chen, 2018), a large body of work has found that the classic distribution of gastric cancer is merely shifting: Since the 1970s, gastric cancer incidence has increased in adults younger than 50 years of age, particularly in females (Anderson et al., 2018; Blaser and Chen, 2018; Kehm et al., 2019). Since the prevalence of *H. pylori* has fallen, it is thought that autoimmune gastritis might be an alternative cause of the rising incidence of gastric cancer in females younger than 50 years of age (Anderson et al., 2018; Blaser and Chen, 2018; Rawla and Barsouk, 2019).

Indeed, the incidence of autoimmune gastritis and autoimmune diseases in general have been increasing in recent decades (Selmi, 2010; Agmon-Levin et al., 2011; Coati et al., 2015; Anderson et al., 2018; Bach, 2018; Dinse et al., 2020). The rise in autoimmune diseases is not fully understood but might be attributable to environmental hygiene, where the population-level frequency of infection is inversely related to the frequency of allergic or autoimmune diseases (Bach, 2018). Autoimmune diseases also commonly display a bias toward females for reasons also not fully understood (Moulton, 2018; Laffont and Guery, 2019). This bias may be attributed to sex hormones (Laffont and Guery, 2019) or prolactin signaling (Borba et al., 2018) and has been observed through X-chromosome dosage in females (Syrett and Anguera, 2019) and in males with Klinefelter’s syndrome (Sawalha et al., 2009). Finally, what classifies autoimmune diseases is not always uniformly defined, and criteria can range from a collection of classic features (female predominance, presence of pathogenic autoantibodies, and response to immunosuppression) to a modified version of Witebsky’s postulates (Hayter and Cook, 2012; Gidwaney et al., 2017).

Despite these challenges, it has long been observed that autoimmune conditions increase a patient’s risk for cancer, a cause-and-effect relationship or the underlying mechanisms remain largely unknown. Recent studies have demonstrated a causal role of autoimmunity in gastric cancer development (Nguyen et al., 2013; Miska et al., 2018). This review will discuss autoimmunity as a potential etiology of cancer, with a focus on type 2 inflammatory signals as a potential tumorigenic factor in the stomach.

## The Advent of Cancer Immunotherapy Highlights the Complex Relationship Between Autoimmunity and Cancer

In the last decade, breakthroughs in cancer immunotherapy have brought substantial survival benefits to some patients by upregulating anti-tumor immunity through immune checkpoint blockade targeting cytotoxic T lymphocyte-associated protein 4 (CTLA4), programmed cell death protein 1 (PD-1), or the programmed cell death 1 ligand 1 (PD-L1) (Couzin-Frankel, 2013; Sun et al., 2018; Kamimura et al., 2019). However, these new therapies can induce autoimmune toxicity even in patients without pre-existing autoimmune conditions (Postow et al., 2018). Additionally, immune checkpoint blockade in cancer patients with pre-existing autoimmune diseases may exacerbate autoimmunity. For example, a retrospective study comparing the use of immune checkpoint inhibition in cancer patients with pre-existing autoimmune diseases and in patients with cancer only reported that the patients with pre-existing autoimmune diseases had significantly higher rates of hospitalization requiring treatment with immunosuppression (Bender et al., 2020). A multicenter study and systematic review similarly found that greater than 70% of cancer patients with pre-existing autoimmune diseases receiving immune checkpoint therapy experienced an exacerbation of those autoimmune diseases or another immune-related adverse event (Abdel-Wahab et al., 2018; Tison et al., 2019).

Patients with autoimmune diseases are commonly treated with immunosuppression and have been suspected to have an increased risk for cancer. For example, anti-tumor necrosis factor therapy was a breakthrough treatment for autoimmune diseases and is indicated to treat rheumatoid arthritis, Crohn’s disease, ulcerative colitis, psoriasis, or ankylosing spondylitis (Monaco et al., 2015). In the 2000s, anti-tumor necrosis factor therapy for rheumatoid arthritis was linked to an increased risk for infection and malignancy (Bongartz et al., 2006). However, those findings have been contradicted by evidence from recent studies: Use of anti-tumor necrosis factor therapy after an initial cancer diagnosis does not influence recurrence or new cancer (Waljee et al., 2020), and patients with inflammatory bowel disease who are treated with this therapy have lower rates of colorectal cancer (Alkhayyat et al., 2020). These findings altogether merit further study of relationship between immunosuppression and the risk for cancer.

Adding to the complex relationship between cancer and autoimmunity is the concept of tumor defense-induced autoimmunity, where an immune response developed against cancer subsequently targets host cells. This idea has been proposed to explain the connection between autoimmunity and cancer in the context of at least the thyroid and dermatomyositis (Aussy et al., 2017; Nagayama, 2018). Thyroid autoimmunity and cancer are highly associated (Ferrari et al., 2020). Nagayama explains that immunogenic tumor antigens may be shared with normal thyroid tissue and the immune response to those antigens also causes autoimmunity (Nagayama, 2018). Patients with dermatomyositis have an increased risk for a variety of cancers (Aussy et al., 2017; Qiang et al., 2017; Gkegkes et al., 2018). Aussy et al. (2017) explain that modification in key dermatomyositis genes may produce neoantigens that can elicit specific anti-tumor responses, but through cross-reactivity or epitope spreading these neoantigens might direct the immune response toward healthy tissue as well.

While major efforts are still needed to understand the various aspects of the intertwined relationship between autoimmunity and cancer (Toomer and Chen, 2014), this review focuses on a new “layer” of their intricate relationship: autoimmunity may have a direct role in triggering tumorigenesis, which has been suggested by the epidemiological trends in cancer and autoimmune diseases as well as experimental evidence.

## Epidemiological Data Reveals an Alarming Rise of Early Onset Gastric Cancer That May Implicate Autoimmunity as a Causal Factor for Cancer Development

At the beginning of the last decade, gastric cancer incidence significantly increased in adults aged 25–39 years from 0.27 to 0.45 per 100,000 person-years, whereas it declined for all older age groups (Anderson et al., 2010). Data from the end of the decade retained the same pattern: The estimated annual percent change in gastric cancer incidence in adults younger than 50 years of age has increased 1.3% annually, whereas it declined by 2.6% annually for older age groups (Anderson et al., 2018). When comparing birth cohorts with individuals born around 1950 as baseline, the incidence rate ratios for gastric cancer increased to 1.36 in individuals born around 1970 and to 1.68 in individuals born around 1985 (Sung et al., 2019).

The increased incidence of cancer in adults younger than 50 years of age has disproportionately increased in females. The incidence of cancer increased 1.15% in 25- to 39-year-old females, increased by 0.46% in males of the same age group, and decreased by 0.31% in 70- to 84-year-old females (Kehm et al., 2019). For gastric cancer specifically, the estimated annual percentage change between 1995 and 2013 in individuals younger than 50 years was 6.0% per year in females and 3.0% per year in males (Anderson et al., 2018). Because autoimmune gastritis also disproportionately affects females and its clinical impact has been increasing, it has been suspected to be a driver of this new trend in gastric cancer (Coati et al., 2015; Anderson et al., 2018; Blaser and Chen, 2018).

Although this review highlights the epidemiology of gastric cancer to discuss the autoimmune etiology of cancer in general, a similar trend has been observed in colorectal cancer. In the United States, the incidence of colorectal cancer in adults aged 20–49 years has increased from 8.6 cases per 100,000 population in 1992 to 13.1 per 100,000 in 2016; similar trends have been reported in Australia, the United Kingdom, and Asia (Stoffel and Murphy, 2020). In Canada prior to 1995, the incidence of colorectal cancer in adults aged 20–29 years was decreasing. From 1995 to 2012 however, the incidence rate has returned to and surpassed historical levels with an estimated annual percentage change of 6.24% in the 20- to 29-year-old cohort (Brenner et al., 2017). The increasing incidence of colorectal cancer in adults younger than 50 years of age might be explained in part by increasing incidence of inflammatory bowel disease (Stoffel and Murphy, 2020). In Olmsted County, Minnesota, the incidence rates of Crohn’s disease and ulcerative colitis in 1970–1979 were 6.9 and 9.2 cases per 100,000 person-years, respectively. In 2000–2010, they increased to 10.7 and 12.2 cases per 100,000 person-years, respectively (Shivashankar et al., 2017). Similarly in Europe, the incidence of Crohn’s disease and ulcerative colitis in 1962 were 1.0 and 6.0 per 100,000 person-years, respectively. In 2010, those incidence increased to 6.3 and 9.8 per 100,000 person-years, respectively (Burisch and Munkholm, 2015). These trends match those that have been seen on a global scale (Kaplan and Ng, 2017). Pediatric inflammatory bowel diseases (patients who are diagnosed younger than 20 years of age) have also increased in populations around the globe (Sykora et al., 2018; Stoffel and Murphy, 2020). In Canada for example, the incidence of inflammatory bowel disease in children younger than 10 years of age increased 7.4% per year between 1994 and 2009 (Ashton et al., 2017). Altogether, autoimmune diseases might play a role in the increasing incidence of gastric and colorectal cancer in adults younger than 50 years of age. Furthermore, the possible tumorigenic effects of autoimmune diseases might stem from common mechanisms related to chronic antigen-specific tissue damage and chronic wound repair, which are discussed in the following sections.

## Chronic Tissue Damage and Wound Healing in Autoimmune Pathogenesis May Reveal New Mechanisms of Tumorigenesis

Hayter and Cook generated a comprehensive list of autoimmune diseases and defined them as having two or more of the following attributes: The adaptive immune response initiates a specific response to the affected organ, autoreactive T cells or autoantibodies are present in the affected organ, autoreactive T cells or autoantibodies transfer the disease to healthy animals, immunization with the autoantigen induces autoimmunity in animal experiments, or that the disease responds to suppression or elimination of autoimmunity (Hayter and Cook, 2012). Some well-defined autoimmune conditions confer an increased risk for cancer (Table 1). Additionally, autoimmune diseases can be associated with increased risk for multiple types of cancers. In these cases, studies often assess a patient’s risk for cancer overall. To reflect this, we report the overall risk for some of the autoimmune diseases listed in Table 1 and refer to their associated cancer as “Multiple.” Dermatomyositis can be associated with nasopharyngeal carcinoma, Barrett’s esophagus, gastrointestinal adenoma, ovarian cancer, and thyroid cancer (Lau et al., 2021; Oldroyd et al., 2021). Rheumatoid arthritis can be associated with lymphoma and lung cancer (Simon et al., 2015). Scleroderma is particularly associated with lung cancer but also with breast, prostate, bladder, gastrointestinal, and hematological cancers (Hill et al., 2003). Systemic lupus erythematosus can be associated with leukemia, Hodgkin’s and non-Hodgkin’s lymphoma, and multiple myeloma. Additionally, this disease can be associated with solid cancers including bladder, cervical, esophageal, gastric, hepatobiliary, lung, non-melanoma skin, oropharyngeal, ovarian, renal, thyroid, and vaginal cancer (Song et al., 2018; Bae et al., 2019; Cobo-Ibanez et al., 2020). Type 1 diabetes can be associated with cancer of the endometrium, kidney, liver, pancreas, and stomach (Carstensen et al., 2016). Altogether, mechanistic understanding of how autoimmunity may lead to cancer development can uncover new mechanisms of tumorigenesis that are relevant to inflammatory carcinogenesis in general.

**TABLE 1:** Patients with autoimmune diseases may have an increased risk of developing cancer.

**Autoimmune Diseases**	**Associated cancer**	**Risk metric (95% CI where available)**	**References**
Autoimmune hepatitis	Hepatocellular carcinoma	CI: 1.1–1.9% per year IR: 3.06 (2.22, 4.23) per 1000py SIR: 23.3 (7.5–54.3)	Carbone and Neuberger, 2014; Sahebjam and Vierling, 2015; Song et al., 2020; Rigopoulou and Dalekos, 2021
Primary biliary cholangitis	Hepatocellular carcinoma	CI: 1.4–12.3% per year IR: 3.4–4.17 cases/1000py RR: 18.80 (10.81, 26.79) SIR: 9.4 (3.04–21.8)	Same as above
Autoimmune gastritis	Gastric adenocarcinoma	RR: 2.84–7 OR: 2.18 (1.94, 2.45)	Bizzaro et al., 2018; Massironi et al., 2019; Song et al., 2019
Crohn’s disease	Colorectal cancer	SIR: 1.7 (1.0, 2.5)	Axelrad et al., 2016; Torres et al., 2017
Ulcerative colitis	Colorectal cancer	SIR: 1.7–14.8	Same as above
Dermatomyositis	Multiple	OR: 14.5 (2.35, 89.3) RR: 2.21 (1.78, 2.77)	Lau et al., 2021; Oldroyd et al., 2021
Rheumatoid arthritis	Multiple	SIR: 1.09 (1.06, 1.13)	Simon et al., 2015
Scleroderma	Multiple	SIR: 1.99 (1.46, 2.95)	Hill et al., 2003
Systemic lupus erythematosus	Multiple	OR: 1.44 (1.33, 1.56) SIR: 1.28–1.37	Song et al., 2018; Bae et al., 2019; Cobo-Ibanez et al., 2020
Type 1 diabetes	Multiple	HR: Males 1.01 (0.98, 1.04), Females 1.07 (1.04, 1.10)	Carstensen et al., 2016

*Literature evidence is summarized from some relatively large-scale studies of cancer risks in patients with well-defined autoimmune conditions. For some autoimmune diseases associated with multiple cancers, the overall cancer risk is listed. Risk metrics combined from multiple studies are shown as a range. CI: cumulative incidence, HR: hazard ratio, IR: incidence rate, OR: odds radio, py: person-years, RR: relative risk or risk ratio, SIR: standardized incidence ratio.*

**FIGURE 1:**
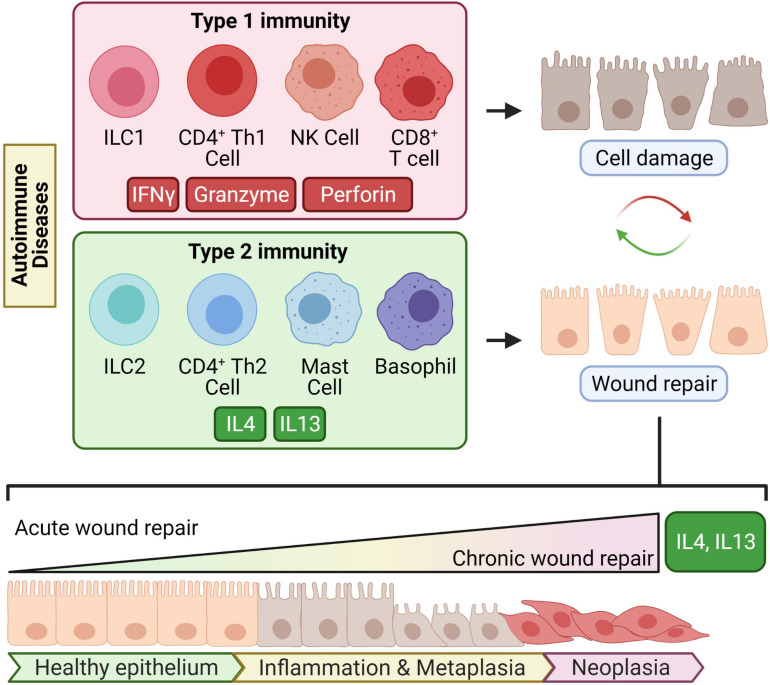
Chronic type 2 immunity in autoimmune diseases may lead to transformation. Type 1 immunity is driven by its key cytokine IFNγ and type 2 immunity by the key type 2 cytokines IL4 and IL13. In several autoimmune disorders, type 1 immunity causes continuous or repetitive cycles of antigen-specific damage to tissues. Type 2 immunity counterbalances type 1 immunity and mediates wound repair. When “chronic would repair” ensues from chronic tissue damage, type 2 cytokines might induce cellular metaplasia and transformation which eventually leads to invasive malignancy.

Chronic damage can be inflicted by autoimmune damage or environmental factors; such factors can include but are not limited to chronic infection or xenobiotics (chemicals or tobacco smoking, for example). These environmental factors can induce chronic cell damage through many mechanisms including inflammation, oxidative stress, direct genotoxicity, or secretion of oncogenic products. These factors can also trigger autoimmune diseases (Agmon-Levin et al., 2011). A notable detail that differentiates autoimmune diseases from environmental factors is antigen specificity-driven tissue damage. Autoimmunity-induced tumorigenesis would begin with cell injury mediated by autoantigen-specific T cells or antibodies driven by any of the three types of immune profiles. Type 1 immunity refers to cell-mediated defense against intracellular pathogens driven by interferon gamma (IFNγ) and CD4^+^ T helper 1 cells (Th1), CD8^+^ cytotoxic T cells, natural killer (NK) cells, and group 1 innate lymphoid cells (ILC1) (Castro et al., 2018a; Kak et al., 2018; Vivier et al., 2018). Type 3 immunity broadly refers to the response orchestrated by IL17 and cells including CD4^+^ T helper 17 cells, and group 3 innate lymphoid cells (Vivier et al., 2018; Yasuda et al., 2019). Type 1 and type 3 immunity are not mutually exclusive entities and can simultaneously cause collateral host cell damage while clearing pathogens (Figure 1).

Type 2 immunity involves CD4^+^ T helper 2 cells (Th2), group 2 innate lymphoid cells (ILC2), follicular T helper cells, basophils, or mast cells that produce the key type 2 cytokines interleukin-4 (IL4) and interleukin-13 (IL13) (Zhu, 2015; Vivier et al., 2018; Barrett and Shalek, 2019; Kumar et al., 2019; Figure 1). In acute settings like infection, type 2 inflammation is well-recognized for its role in wound healing, B cell differentiation, and antibody production (Lloyd and Snelgrove, 2018). The wound-healing capacity of type 2 inflammation is presumably evolved to repair the collateral tissue damage from destructive type 1 immunity mounted against infectious agents.

In chronic settings, the effects of type 2 immunity are not as “benign,” and “chronic wound healing” can go awry. For example, type 2 cytokines are common mediators of fibrosis in chronic diseases (Liu et al., 2012; Passalacqua et al., 2017; Nguyen et al., 2020). Beyond fibrosis, type 2 immunity can lead to abnormal differentiation of cellular lineage and tumorigenesis (Figure 1); this has been supported with experimental evidence from mouse models of CTLA4 insufficiency (Miska et al., 2018).

## Ctla4 Insufficiency and Other Models of Autoimmune Gastritis Provide Experimental Evidence of Autoimmunity-Driven Tumorigenesis

**FIGURE 2:**
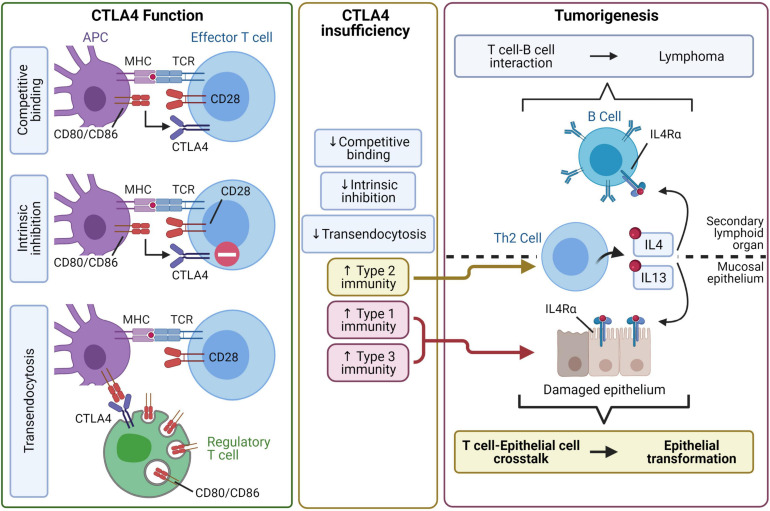
CTLA4 insufficiency may lead to autoimmune tumorigenesis through type 2 inflammatory pathways. Professional antigen-presenting cells (APC) activate T cells by providing co-stimulation through antigen-loaded MHC and CD80/CD86. CTLA4 negatively regulates of T cell activity through at least three mechanisms. CTLA4 can bind to CD80/CD86 with high affinity and sequester CD80/CD86 from CD28-mediated co-stimulation. Alternatively, CTLA4 binding to CD80/CD86 may initiate intrinsic inhibitory signaling in T cells. Furthermore, CTLA4, especially with constitutive expression by regulatory T cells, binds to CD80/CD86 and removes them from the APC by transendocytosis. CTLA4 insufficiency impairs the regulation of T cells, leading to dysregulated type 1, type 2, and type 3 immunity. The proposed pathway for tumorigenesis focuses on dysregulated IL4/IL13-driven type 2 immunity. In secondary lymphoid organs, dysregulated Th2 cell-B cell interaction may lead to the development of lymphoma. In mucosal epithelia, type 1 and type 3 immunity may inflict epithelial damage while type 2 immunity initiates a Th2 cell-epithelial cell crosstalk that potentially leads to epithelial cell transformation.

The possible link between autoimmunity and cancer suggested by epidemiological findings is also strongly suggested in clinical studies of patients with CTLA4 insufficiency. CTLA4 is a key checkpoint for maintaining immune tolerance and regulating T cell activation. A few mechanisms by which this occurs have been proposed. When CTLA4 is expressed on an effector T cell, it might competitively bind to CD80 and CD86 on antigen-presenting cells and either simply block co-stimulation or actively elicit an intrinsic inhibitory signal. Alternatively, regulatory T cells expressing CTLA4 may bind to and remove CD80/CD86 from antigen-presenting cells through transendocytosis (Rowshanravan et al., 2018; Figure 2). Patients can develop CTLA4 insufficiency by inheriting mutations in one allele of *CTLA4* or from polymorphisms, several of which exist in the general population. Independent studies have found a modest association of gastric cancer with some of the polymorphic alleles at the *CTLA4* promoter and exon 1 regions (Hadinia et al., 2007; Hou et al., 2010), and those alleles are known to cause reduced CTLA4 expression (Ligers et al., 2001; Anjos et al., 2002; Wang et al., 2002).

As one would expect based on the key role of CTLA4 in immune tolerance, patients with CTLA4 haploinsufficiency present with various autoimmune conditions including autoimmune enteropathy, atrophic gastritis, and hematologic autoimmune conditions (Schubert et al., 2014; Egg et al., 2018; Schwab et al., 2018). Strikingly, these patients have an increased risk for lymphoma or gastric cancer. The patients who developed gastric cancer had a median age of onset of 34 years and long-standing histories of atrophic gastritis, autoimmune enteropathy, and/or pernicious anemia (Egg et al., 2018; Schwab et al., 2018).

Of note, pernicious anemia, which results from autoimmune damage of gastric mucosa, has long been known as a risk factor for gastric cancer development (Hsing et al., 1993). This condition has been modeled in mice, which undergo epithelial transformation to gastric metaplasia (Nguyen et al., 2013). This model has suggested a link between autoimmunity and tumorigenesis, where administration of IL27, which may inhibit all three types of immunity (Yoshida and Hunter, 2015; Tait Wojno et al., 2019), blocked the formation of metaplasia (Bockerstett et al., 2020). The idea that type 2 inflammation induces epithelial transformation has been confirmed in an animal model system mimicking human CTLA4 insufficiency. These CTLA4-insufficient mice retain the increased risk for cancer development seen in humans and spontaneously develop parietal cell atrophy, gastric metaplasia, and cancer (Miska et al., 2018). In this study, Miska et al. (2018) show that blocking the two key type 2 cytokines IL4 and IL13 abrogates tumorigenesis. A third group that studies drug-induced stomach injury and metaplasia found that ILC2s, which are activated by and perpetuate type 2 inflammation, are required for the development of epithelial metaplasia (Meyer et al., 2020).

Based on these data, a possible pathophysiologic pathway is visualized in Figure 2. First, CTLA4 insufficiency may lead to T cell proliferation and dysregulated immune responses. Th2 dysregulation in secondary immune tissues like the spleen and lymph nodes might facilitate an aberrant T cell-B cell interaction leading to lymphoma. Interestingly, IL13 and its major signaling mediator signal transducer and activator of transcription (STAT) 6 are known to play roles in B and T cell lymphomas (Fior et al., 1994; Gaydosik et al., 2020). Of note, constitutive expression of CTLA4 is essential for the regulatory role of regulatory T (T_reg_) cells. This has been demonstrated in models of conditional CTLA4 knockout in Foxp3^+^ cells, where complete elimination of CLTA4 abrogates T_reg_ suppressive activity *in vitro* and *in vivo* (Wing et al., 2008). Along this line, the CD4^+^CD25^+^ cells of patients with CTLA4 haploinsufficiency show reduced suppressive activity *in vitro* (Kuehn et al., 2014; Schubert et al., 2014). However, reduction of CTLA4 expression to approximately 40% of normal levels in a CTLA4 knockdown model does not compromise the function of CD4^+^CD25^+^Foxp3^+^ T_reg_ cells. Rather, their suppressive effects are increased due to enhanced formation of effector memory T_reg_ cells (Devarajan et al., 2014). In the model of gastric tumorigenesis caused by CTLA4 insufficiency, we found that the defect caused by CTLA4 insufficiency did not reside in T_reg_ cells but rather in the non-T_reg_ compartment (Miska et al., 2018). Infiltration into organs with a mucosal epithelial lining, such as in the stomach, can provide the setting for interaction between immune and epithelial cells. More specifically, this interaction may consist of a repeated cycle of epithelial cell damage induced by autoimmune type 1 inflammation and epithelial cell repair mediated by type 2 inflammation. It is in this setting that chronic type 2 inflammation may eventually result in epithelial transformation.

In all, multiple independent groups with different mouse models have gathered robust evidence for type 2 inflammation as a cause of tumorigenesis. The next pertinent questions will be exactly how this process occurs. The mechanisms by which type 2 inflammation induces tumorigenesis can include epigenetic shifts in epithelial cells, changes in gene expression and cell fate, and creation of reactive oxygen species (ROS) and oxidative damage by a type 2 inflammation-driven pathway.

## Type 2 Inflammation-Induced Epigenetic Changes May Underlie Autoimmune Tumorigenesis

**FIGURE 3:**
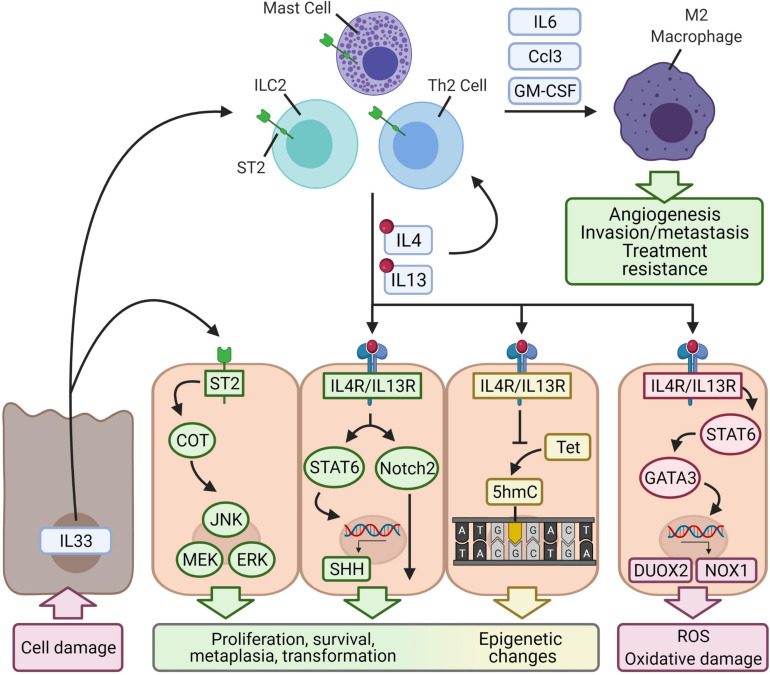
Proposed mechanisms of cellular transformation by type 2 inflammation. Autoimmunity causes antigen-specific tissue damage and may lead to the release of IL33, which is constitutively expressed and acts as an alarm signal when released into the extracellular environment. IL33 binds to the ST2 receptor expressed on epithelial cells and induces Cancer Osaka Thyroid (COT), which in turn activates pathways that regulate cell proliferation (JNK, MEK/ERK). IL33 also binds to ST2 expressed on ILC2s, Th2 cells, or mast cells and activates them. These cells secrete the type 2 cytokines IL4 and IL13, which reinforce type 2 immunity and bind to IL4R/IL13R expressed by epithelial cells. Downstream activation of STAT6 upregulates Sonic hedgehog (SHH), a regulator of embryonic genes. IL4R/IL13R may also activate Notch2, which regulates cell fate and differentiation. IL4-, IL13-, and IL33-induced changes to gene expression are complemented by epigenetic changes. Type 2 signaling in epithelial cells downregulates a ten-eleven translocation enzyme (Tet), which converts methylated cytosine to 5-hydroxymethylcytosine (5hmC), a common cancer epigenetic marker. Altogether, type 2 signaling may induce transformation of epithelial cells. IL4R/IL13R-STAT6-GATA3 signaling also produces reactive oxygen species by upregulating NADPH oxidase homologs DUOX2 and NOX1, which generate reactive oxygen species. The type 2 immune cells also secrete granulocyte-monocyte colony-stimulating factor (GM-CSF), chemokines, and IL6 to recruit M2-polarized macrophages. These macrophages are well-known to mediate tumor progression, angiogenesis, invasion/metastasis, and resistance of cancer to treatment.

Epigenetic perturbations have been increasingly documented in contributing to the initiation and progression of various cancers (Kanwal et al., 2015) including stomach (Samadani et al., 2019), lung (Liu and Zhao, 2019), colon (Jung et al., 2020), breast (Pasculli et al., 2018), and prostate cancer (Wu et al., 2015; Tzelepi et al., 2020). In general, one of the most recognized epigenetic changes in tumorigenesis is the methylation of genomic DNA at the 5 position of cytosine (5mC) (Jones, 2012). Cancer research was accelerated by the discovery of the tumor-associated loss of 5-hydroxymethylcytosine (5hmC), an epigenetic marker that is easier to quantify (Lian et al., 2012). Among many other signals that trigger epigenetic changes, evidence has emerged suggesting that type 2 inflammation can induce potentially tumorigenic epigenetic changes. In the CTLA4 insufficiency model, IL4/IL13 signaling was associated with a downregulation of both ten-eleven translocation methylcytosine dioxygenase (Tet) 3 and 5hmC (Miska et al., 2018). The Tet proteins impact the epigenetic profile by participating in active DNA demethylation by oxidizing 5mC into 5hmC, 5-formylcytosine, or 5-carboxylcytosine. 5-formylcytosine and 5-carboxylcytosine undergo base-excision repair and are replaced by unmodified cytosine (Shi et al., 2017). 5hmC is involved in DNA methylation and epigenetic reprogramming driving transformation of multiple cancer types (Thomson and Meehan, 2017). Because type 2 cytokines appear to participate in 5hmC regulation, they may contribute to an epigenetic-driven mechanism of cellular reprogramming to tumorigenesis (Figure 3).

Type 2 inflammation-driven metaplastic differentiation and malignant transformation at the epigenetic level has also been observed in chronic rhinosinusitis with nasal polyposis (May and Fung, 2015; Barrett and Shalek, 2019). Chronic rhinosinusitis is also associated with goblet cell metaplasia, and recent single-cell RNA sequencing experiments revealed that epithelial basal cells, which give rise to ciliated cells and goblet cells in the airway, also undergo metaplasia and dysplasia (Barrett and Shalek, 2019). Specifically, a subset of epithelial basal cells expresses IL33 and thymic stromal lymphopoietin, leading to the activation of mast cells and ILC2s which secrete type 2 cytokines. Ultimately, this pathway promotes an epigenetic shift that leads to an IL4/IL13 gene expression signature in basal cells associated with Wnt-related wound repair, basal cell hyperplasia, and dysplasia (Barrett and Shalek, 2019; Bankova and Barrett, 2020). Specifically, IL4 and IL13 upregulates expression of *CTNNB1* (β-catenin) and downstream Wnt target genes. Therefore, chronic IL4/IL13 signaling may result in persistent expression of the Wnt/β-catenin pathway.

## Intracellular Signaling by Type 2 Cytokines May Cause Transcriptional Reprogramming and Alter Cell Fate

While it remains largely unknown how exactly type 2 cytokines might promote cellular transformation, evidence gathered from some chronic inflammatory conditions offers some clues on cellular proliferation and lineage perturbation. In clinical pathology, chronic airway diseases such as chronic bronchitis, asthma, and cystic fibrosis are known to lead to goblet cell metaplasia, a proliferation of goblet cells that mediates mucus hypersecretion (Danahay et al., 2015; Pezzulo et al., 2019; Wang et al., 2020b). Studies have suggested that type 2 inflammation through IL4 and/or IL13 induces goblet cell metaplasia. Wang et al. (2020b) demonstrate that IL4 and IL13 via STAT6 mediate transition of allergic bronchial pathology to goblet cell metaplasia by upregulating the expression of Sonic hedgehog, an embryonic developmental gene that regulates cell cycle and proliferation targets like cyclin-D2, cyclin-E, and N-myc (Skoda et al., 2018). Danahay et al. (2015) demonstrate that IL13 induces Notch2 signaling, and this pathway biases basal cell differentiation toward a goblet cell fate and away from one of a ciliated cell. Pezzulo et al. (2019) show that geldanamycin, an inhibitor of heat shock protein 90, blocked IL13- and IL17-induced goblet cell metaplasia. They hypothesize that the mechanism of action involves TGF-β and ETS homologous factor and/or epidermal growth factor receptor signaling through ErbB. In this context, type 2 cytokines may impact the expression of genes related to proliferation, survival, metaplasia, and ultimately transformation; these changes in gene expression might work in tandem with the epigenetic changes (Figure 3).

## Type 2 Inflammation May Promote Tumorigenesis by Producing Ros

It is well-accepted that mutagenic hits play an essential role in malignant transformation (Knudson, 1971). Then, can type 2 inflammation inflict genetic damage and thus promote tumorigenesis? Indeed, type 2 inflammation can inflict oxidative damage through the induction of NADPH oxidase homologs, which include dual oxidases (DUOX) and NOX enzymes. DUOX1 and DUOX2 reduce molecular oxygen into superoxide and hydrogen peroxide (De Deken et al., 2014), two examples of ROS, and NOX enzymes utilize NADPH to generate superoxide (Bedard and Krause, 2007). The tumorigenic effects of ROS are well-understood and mediated at least in part by genotoxicity (Kay et al., 2019). Briefly, ROS can directly oxidize DNA, leading to nucleotide alterations (deamination, transitions, and transversions, for example) or single- and double-stranded breaks.

Type 2 inflammation has been shown to induce expression of the specific NADPH oxidase homologs DUOX2 and NADPH oxidase 1 (NOX1), and the subsequent production of ROS might contribute to type 2 inflammation-driven tumorigenesis through ROS-related mechanisms (Figure 3). In human pancreatic and colon cancer cell lines, IL4-STAT6 signaling has been shown to increase DUOX2 expression, and this effect synergizes with IL17A (Wu et al., 2019). The upregulation of this enzyme correlates with increased production of hydrogen peroxide. In addition to their pro-fibrotic effects in the lung, IL4 and IL13 have also been shown to mediate chronic oxidative damage and inflammation following lung irradiation: Irradiated rats showed increases in IL4, IL4Rα1, and DUOX2 along with increased infiltration of macrophages, lymphocytes, and mast cells (Aliasgharzadeh et al., 2019). IL4, when its receptor is present, has been shown to increase NOX1 levels through JAK1/STAT6 signaling (Liu et al., 2017). STAT6 subsequently activates GATA3, which then binds to the NOX1 promoter and induces expression.

## Il33 Is Triggered by Autoimmune Tissue Damage and Potentially Initiates Type 2 Inflammation

Although it is unknown exactly how autoimmune tissue damage may trigger type 2 inflammation and tumorigenesis, IL33 could be a major player. IL33 is constitutively expressed in epithelial cells, endothelial cells, and fibroblasts and can be induced in hematopoietic cells like mast cells, macrophages, and neutrophils (Afferni et al., 2018; Eissmann et al., 2020). Under physiologic conditions, IL33 localizes to the nucleus where it can regulate gene expression by binding to histones or influencing histone deacetylase activity (Afferni et al., 2018; Larsen et al., 2018). Upon cell damage and necrosis, IL33 is released into the extracellular environment and acts as an alarmin by binding to the ST2 receptor. The ST2 receptor is expressed in most hematopoietic cells, particularly T_reg_ cells, ILC2 cells, and mast cells (Afferni et al., 2018; Figure 3). This pathway upregulates type 2 inflammation by skewing naïve CD4^+^ T cells toward Th2 or CD4^+^ T helper 9 cell differentiation, triggering ILC2 cells to secrete IL5 and IL13, activating mast cells, and driving M2 polarization of macrophages (Afferni et al., 2018; Eissmann et al., 2020).

IL33 may directly promote epithelial cell tumorigenesis, a pathway that has been suggested by a study of breast cancer (Kim et al., 2015). IL33 and ST2 were found to activate a Cancer Osaka Thyroid oncogene, which in turn activated the MEK-ERK, JNK, and STAT3 pathways, all of which play roles in cell survival and proliferation (Roberts and Der, 2007; Weston and Davis, 2007; Elinav et al., 2013; Figure 3).

On the other hand, the role of IL33 in tumorigenesis may be mediated by type 2 inflammation through an IL33/ILC2/IL13 pathway (Figure 3). One study investigating the link between IL33 and the development of cholangiocarcinoma found that daily injections of IL33 increased the size and thickness of extrahepatic bile ducts and induced metaplastic changes (Li et al., 2014). Repeating the procedure in a pathologic setting of a constitutively active Akt and Hippo pathway [which modulates immunity and the tumor microenvironment (Taha et al., 2018)] facilitates malignant transformation. The group attributed the hyperplastic response to ILC2 cells and their secretion of IL13. Along this line, a recent study of drug-induced metaplasia in the stomach found ILC2s to be the coordinators of damage response and epithelial transformation (Meyer and Goldenring, 2018). Here, IL33 is required for the activation of ILC2s, which develop a unique metaplasia-related transcriptional profile (determined by single cell RNA sequencing) associated with the expression of the *Csf2* gene (which encodes granulocyte-macrophage colony-stimulating factor), IL receptor subunit genes *Il2rb* and *Il4ra*, and an immune checkpoint gene *Pd1*. These ILC2s subsequently secrete and are the major source of IL13.

Furthermore, an IL11/IL33/mast cell/macrophage pathway has also been shown to be required in the early stages of gastric cancer development (Eissmann et al., 2019; Figure 3). Mast cells appear in greater density in gastric cancer and determine macrophage infiltration, the presence of which forms the rate-limiting factor for tumor growth and establishment of microvasculature (Eissmann et al., 2019). The role of IL33 in this pathway is different from the one described above. IL11, possibly secreted from subepithelial myofibroblasts or tumor-associated endothelial cells, signals through STAT3 and stimulates gastric tumor epithelium to release IL33. This tumor-derived IL33 binds to ST2 receptors on mast cells, which become activated and release macrophage-attracting factors *Csf2*, *Ccl3*, and *Il6*. Infiltrating macrophages then coordinate their well-known pro-tumor effects, such as increased angiogenic *Vegfa* expression (Eissmann et al., 2019).

## Immune-Epithelial Crosstalk Through Dysregulated Type 2 Inflammatory Signals Facilitates a Tumorigenic Microenvironment

In the setting of chronic tissue damage, the multiple pathways covered in previous sections may act in an integrated manner leading to type 2 inflammation-driven tumorigenesis. In brief, type 2 inflammatory tumorigenesis may start with a source of chronic cell damage, and injured epithelial cells release IL33 as an alarmin. IL33 induces the emergence of various type 2 immune cells, most notably ILC2s and mast cells. The secretion of type 2 cytokines may have two effects: (1) the type 2 environment is perpetuated, as type 2 cytokines themselves lead to the activation and differentiation of type 2 immune cells; (2) type 2 cytokines initiate global transcriptional changes within the injured epithelium.

IL33 may also enhance a type 2 microenvironment once the tumor is established. IL11 may trigger tumor cells to release IL33, potentially shifting it from an alarmin to an actively secreted product. Furthermore, ILC2s and mast cells may both release macrophage attractants leading to the infiltration of M2 macrophages. The presence of M2 macrophages might then determine the progression of cancer, as the pro-tumor role of M2 macrophages in established cancer is well-documented. These effects include epithelial-mesenchymal transition (Yao et al., 2018a), invasion/metastasis (Binnemars-Postma et al., 2018; Little et al., 2019), and cancer cell resistance to various treatments like etoposide (Genin et al., 2015), tamoxifen (Li et al., 2020), or sorafenib (Dong et al., 2019).

This proposed pathway raises some considerations. The epigenetics-driven mechanism of transformation implies that type 2 inflammation is capable of mediating malignant transformation in the absence of mutations to oncogenes or tumor suppressor genes. Indeed, some cancers can have relatively lower mutational burdens, including gastric cancer (Chalmers et al., 2017; Salem et al., 2018). On the other hand, type 2 cytokines might also upregulate ROS production. ROS not only contributes to tumorigenesis by damaging DNA and DNA repair enzymes but may additionally cause more cell damage and further increase the type 2 immune response. However, the weight of its contribution to tumorigenesis in the type 2 inflammation context is not known, but it may be smaller than expected when considering the relatively lower mutational burden in gastric cancer (Chalmers et al., 2017). Conversely, not all patients with autoimmune or chronic diseases develop cancer, and the effect of ROS can be at least one factor explaining the stochastic nature of type 2 inflammatory tumorigenesis.

The key implication of the IL33/ST2 pathway is that any cause of cell damage leads to the release of IL33. This pathway can possibly explain how a heterogeneous group of diseases like the autoimmune diseases can converge on similar pathways leading to tumorigenesis. While autoimmune diseases are commonly classified by the branch of immunity that drives the immunopathology, the IL33/ST2/Type 2 inflammation pathway could be a major player, or at least a common element, in the reparative response to cell injury. Release of IL33 in the setting of acute cell damage induces an acute regenerative response, and prolonged cycles of cell damage may induce a progression from repetitive regeneration to metaplastic repairing to eventual transformation.

## Type 2 Inflammation May Be a Shared Process of Tumorigenesis Driven by Autoimmunity and Other Chronic Inflammatory Conditions

The association of type 2 inflammation with tumorigenesis in humans is not limited to autoimmune disorders but also plays roles in a variety of conditions including infection and autoinflammatory conditions. A recent cohort study found that patients who developed hepatocellular carcinoma despite maintaining a sustained virological response against hepatitis C virus were linked to increased circulating levels of IL13 (Macek Jilkova et al., 2020). These patients were matched to controls who did not develop hepatocellular carcinoma, and IL13 was found to be the only strong factor associated with its development. These findings are consistent with two studies from one group indicating that a shift from a Th1- to a Th2-dominant response in patients with hepatitis C virus-induced liver cirrhosis may be responsible for subsequent development of hepatocellular carcinoma (Matsui et al., 2008; Kogame et al., 2016).

Chronic rhinosinusitis is associated with an increased risk for head and neck cancer including nasopharyngeal carcinoma (Riley et al., 2016; Wu et al., 2017; Xia et al., 2019). Chronic rhinosinusitis with nasal polyposis is a subtype that is driven by type 2 inflammation (Barrett and Shalek, 2019), and a 2014 retrospective cohort study split patients with rhinosinusitis into subcohorts of chronic sinusitis only, nasal polyposis only, or both (Tsou et al., 2014). The study found that all three groups had an increased risk of developing nasopharyngeal carcinoma compared to a without-rhinosinusitis control group and additionally found that the nasal polyposis-only subcohort had a greater risk than the remaining two subcohorts. Cohort studies examining nasal polyposis alone have found significant associations with head and neck cancer (Pourang et al., 2018; Kim et al., 2019).

Atopic dermatitis is an immune-driven chronic inflammatory skin condition with epithelial barrier dysfunction (Avena-Woods, 2017; Furue et al., 2017). Type 2 effector cells, specifically ILC2s and Th2 cells (Leyva-Castillo et al., 2020), play a part in driving pathology (Avena-Woods, 2017; Furue et al., 2017; Gavrilova, 2018). Affected epithelial cells can induce type 2 immunity through secretion of thymic stromal lymphopoietin and IL33 (Gavrilova, 2018). Atopic dermatitis has been linked with increased risk for cancers including basal cell and keratinocyte carcinoma, kidney cancer, and colorectal cancer (Gandini et al., 2016; Chou et al., 2020; Wang et al., 2020a). Treatments for atopic dermatitis that target type 2 inflammation are already in place, and more are being investigated. For example, blocking IL4Rα with dupilumab is an effective treatment (Blauvelt et al., 2017; Deleanu and Nedelea, 2019). The anti-IL13 therapies lebrikizumab and tralokinumab for moderate-to-severe atopic dermatitis have been undergone phase 2 or 3 clinical trials (Guttman-Yassky et al., 2020; Silverberg et al., 2020).

## Type 2 Inflammatory Pathways Unveiled in Studies of Autoimmune Tumorigenesis May Provide New Targets to Improve Cancer Immune Therapy

Immune checkpoint inhibition became a breakthrough cancer treatment because of unprecedented efficacy in treating metastatic melanoma, non-small cell lung cancer, colorectal cancer, and hepatocellular carcinoma (El Dika et al., 2019; Zemek et al., 2020). However, these prominent examples are unfortunately outweighed by the number of patients who see modest survival gains, no clinical benefit, or develop resistance (Li et al., 2019; Kalbasi and Ribas, 2020; Zemek et al., 2020). In the CTLA4 insufficiency model, treatment with anti-IL4-antibody and knockout of IL4Rα did not prevent inflammation but severed its link with the initiation of tumorigenesis (Miska et al., 2018), suggesting that therapies targeting type 2 inflammation may be useful in promoting anti-cancer immunity or cancer prevention as well. This idea could be further supported by other models of gastric injury which have found that ILC2s are key mediators of a metaplastic response or that administration of IL27, an inhibitor of type 2 and other types of immunity (Yoshida and Hunter, 2015; Tait Wojno et al., 2019), protects against metaplasia (Bockerstett et al., 2020; Meyer et al., 2020).

Type 2 immunity is a well-known player in already-established cancer. Elevated levels of IL4, IL13, or their receptors have been associated with poorer patient outcomes such as cancer progression, recurrence, or reduced survival. Some examples where this is the case include but are not limited to colon cancer (Barderas et al., 2012; Delgado-Ramirez et al., 2020), clear cell renal cell carcinoma (Chang et al., 2015), bladder cancer (Joshi et al., 2014), pleural mesothelioma (Burt et al., 2012), ovarian cancer (Kioi et al., 2005), and soft tissue sarcomas (Kim et al., 2021). Type 2 immunity has already been investigated as a target for treatment. Blocking M2 polarization of macrophages with astragaloside IV (an herbal compound) or imatinib (a tyrosine kinase inhibitor currently used in the treatment of chronic myelogenous leukemia) has been demonstrated experimentally to block cancer cell metastasis *in vitro* (Xu et al., 2018; Yao et al., 2018b). Cancer cells highly expressing IL4 and IL13 receptors can be targeted by interleukins conjugated to *Pseudomonas* exotoxin or targeted doxorubicin liposomes (Kawakami et al., 2002a,b; Kioi et al., 2005; Suzuki et al., 2015).

A new potential treatment modality for cancer could be dupilumab, an antibody targeting IL4Rα, a receptor subunit for IL4 and IL13. As discussed above, dupilumab is used to treat moderate-to-severe atopic dermatitis (Frampton and Blair, 2018), and it has been tested for safety and efficacy in the treatment of other type 2 immunity-driven diseases like chronic rhinosinusitis with nasal polyps (Bachert et al., 2019) and moderate-to-severe uncontrolled asthma (Castro et al., 2018b). As one would speculate, dupilumab would probably not be indicated for T-cell lymphoma or other types of malignant cells, where intrinsic or extrinsic type 2 cytokines trigger inhibitory signals of cellular proliferation. On the other hand, it is appealing to test the safety and efficacy of combining dupilumab with checkpoint blockade therapy to treat cancers of epithelial origin that express high levels of IL4Rα in tumor cells.

Some cancer treatments can increase the risk for future cancer, such as cyclophosphamide’s association with bladder cancer. A new key implication arises when incorporating the reviewed mechanisms of type 2 inflammation-mediated tumorigenesis and the current strategies targeting immune checkpoints: Anti-CTLA4 therapies can mimic a CTLA4 insufficiency and potentially initiate a cascade of type 2 inflammatory signaling that results in tumorigenesis. It remains to be seen whether anti-CTLA4 treatment may confer a secondary cancer risk in the very long term, and whether type 2 inflammation has a role in that regard.

## Conclusion and Future Directions

The rise in incidence of autoimmune diseases parallels the rise in incidence of their respective cancers, such as autoimmune gastritis and gastric cancer or inflammatory bowel diseases and colon cancer. The increased incidence of these cancers is especially conspicuous in adults younger than 50 years of age and, in the case of gastric cancer, the increase disproportionately affects females (Anderson et al., 2018; Kehm et al., 2019). This epidemiological evidence suggests that autoimmunity might be a cause of cancer. This link became more apparent in studies of patients with CTLA4 haploinsufficiency, who present with a complex syndrome including autoimmunity and an increased risk for gastric cancer and lymphoma (Egg et al., 2018; Schwab et al., 2018). Studies using a mouse model mimicking the CTLA4 insufficiency in these patients, along with the findings from other groups using different animal model systems, have gathered robust evidence for a causal role of autoimmunity in tumorigenesis (Nguyen et al., 2013; Miska et al., 2018). In particular, autoimmunity may initiate organ-specific cell damage and trigger a reparative response mediated by type 2 immunity. This type 2 immune response, however, might also induce epithelial cell metaplasia, transformation, and eventual progression to invasive malignancy (Miska et al., 2018).

Type 2 immunity might induce epithelial cell transformation through multiple mechanisms. It has been shown to induce epigenetic shifts in epithelial cells, and this is accompanied by type 2 cytokine-mediated alterations in transcriptional profile and cell fate. IL33 may be a key cytokine that initiates a self-perpetuating, type 2-predominant, pre-metaplastic microenvironment that facilitates type 2 immune cell and epithelial cell crosstalk. Importantly, since IL33 initiates these effects following cell damage (Afferni et al., 2018; Larsen et al., 2018), it may explain how a wide variety of autoimmune and non-autoimmune inflammatory conditions can have a similar outcome of tumorigenesis; on the other hand, repetitive cycles of antigen-specific damage may further enhance IL33 and ensuing inflammatory signals in autoimmune conditions.

Besides its effect on *de novo* tumorigenesis, type 2 immunity is well-known as an antagonist of antitumor immunity in established tumors, and the pro-tumor effects of M2 macrophages or myeloid-derived suppressor cells are associated with poor patient outcomes like metastasis (Chanmee et al., 2014; Veglia et al., 2021). With the new knowledge that type 2 immunity can initiate *de novo* tumorigenesis, anti-type 2 immunity therapies such as dupilumab may hold promise not only as a new cancer treatment but also as a new preventative measure of cancer development in at-risk patients.

Cancer will continue to be a major disease and will likely continue increasing its burden on humanity for foreseeable future. Indeed, the World Cancer Report 2014 predicted a dramatic increase of cancer incidence in the next 20 years. While this prediction is based largely on the rapid growth of aging populations, it is now apparent that the overall cancer burden can also be exacerbated by the recently uncovered trend of increasing cancer incidence in young adults. As the report warned, it is impossible to “treat our way out of the cancer problem,” and it is critical to develop effective measures of cancer prevention to avert this dangerous trend. At the very least, mechanistic understanding of inflammatory tumorigenesis, a common theme underlying many types of cancers, in all its forms may lead new approaches in cancer prevention. If the emerging relationship between autoimmunity-derived type 2 inflammation and tumorigenesis holds true, the prevention and treatment of some autoimmune diseases could coincide with the prevention of some types of cancer and the subversion of its rising incidence in young adults.

## Author Contributions

CL researched and wrote the manuscript. ZC conceived the thematic focus and overall direction, reviewed and edited the manuscript. Both authors designed the figures.

## Conflict of Interest

The authors declare that the research was conducted in the absence of any commercial or financial relationships that could be construed as a potential conflict of interest.

## Abbreviations

*H. pylori*: *Helicobacter pylori*
PD-1: programmed cell death protein 1
PD-L1: programmed cell death 1 ligand 1
IFNγ: interferon gamma
Th1: CD4^+^ T helper 1
NK cell: natural killer cell
ILC1: group 1 innate lymphoid cell
Th2: CD4^+^ T helper 2
ILC2: group 2 innate lymphoid cell
STAT: signal transducer and activator of transcription
5mC: 5-methylcytosine
5hmC: 5-hydroxymethylcytosine
Tet: ten-eleven translocation methylcytosine dioxygenase
DUOX: dual oxidase
ROS: reactive oxygen species
NOX1: NADPH oxidase 1.
